# How to Treat T Cell Mediated Rejection? -A Call for Action

**DOI:** 10.3389/ti.2024.12621

**Published:** 2024-04-18

**Authors:** Klemens Budde

**Affiliations:** Department of Nephrology, Charité Universitätsmedizin Berlin, Berlin, Germany

**Keywords:** survey, ESOT, T cell mediated rejection (TCMR), European practices, treatment landscape

Over the last decades early rejection rates decreased, the majority of T cell mediated rejections (TCMR) respond to treatment [1, 2], and Banff borderline category is the most frequent finding in early biopsies questioning the clinical relevance of TCMR today. However, severe TCMR may cause nephron loss and inferior outcomes and is associated with development of donor-specific antibodies (DSA) and ABMR [3–5]. Recent evidence suggests that TCMR contributed to 34% of graft losses, compared to 31% due to ABMR [6]. The fact that a single rejection episode, which responds to treatment is not associated with worse graft outcome [2] supports the need for effective TCMR therapies. Despite initial treatment response, 39% of patients have persistent borderline or TCMR after anti-rejection therapy [4] and ongoing inflammation is associated with inferior outcomes and sensitization [4, 5]. In addition, anti-rejection therapy has many side effects causing significant morbidity and even mortality [7]. Thus, there remains a high unmet medical need for better and less toxic treatments for TCMR.

Given the importance of rejection since the early days of transplantation it is surprising to find only sparse high-quality evidence for anti-rejection therapy [4, 7–9]. The use of steroids and lymphocyte depleting agents for anti-rejection therapy dates back to the sixties with approval before the introduction of the Banff classification, when rejection rates were around 50%. Despite low-level evidence all transplant physicians have made personal experiences that steroids and anti-lymphocyte preparations are very effective in the treatment of TCMR, which might explain the lack of randomized trials for TCMR therapy under tacrolimus and mycophenolate. Thus, our current approach, although successful, is outdated as all previous evidence comes from an era with a different maintenance immunosuppression, different organ quality, limited ability to detect sensitization, and even without a clear differentiation between TCMR and ABMR.

In order to advance the field, the transplant community needs to re-focus on TCMR, to describe current standard of care for diagnosis and treatment and to define relevant treatment goals. The paper of the ESOT working group [9] in the current issue of the journal is an important step in this direction. This manuscript reports the results of a survey of 129 experienced European kidney transplant professionals (mainly nephrologists) on the diagnosis and treatment of TCMR and borderline lesions. For TCMR diagnosis European experts rely on traditional biomarkers and biopsies classified according to the most recent Banff classification. Protocol biopsies are performed in 57.5% of centers, although only 36% perform protocol biopsies in all patients.

Contrary to US [10], and similar to Canada [11], treatment for TCMR appears rather homogeneous across Europe [9]. TCMR and borderline changes in indication biopsies are treated with a steroid pulse and depending on the severity of rejection followed by lymphocyte depleting agents as second line treatment. Treatment of rejection is more heterogeneous in protocol biopsies, especially for borderline changes in whom only 62% receive high dose steroids. European experts agree to assess treatment effect early, however timing and assessment of response differed. Most respondents rely on the evolution of renal function within 1–4 weeks, although a large proportion considered a second biopsy important to assess efficacy and steroid resistance.

The excellent survey and the straightforward analysis provide crucial information on the common practices in Europe for diagnosis and treatment of TCMR. Together with surveys from US and Canada [9–11] the data are extremely helpful for clinical care, research, policy making, regulatory authorities, pharma industry, and future clinical trials. The survey highlights the need for standardized definitions, e.g., for steroid refractory rejection or treatment response. ESOT, together with other stakeholders could start an initiative for such standardized definitions for use in clinical practice, research and regulatory demands extending previous publications [1, 12]. Updated guidelines for follow-up biopsies, a more precise description of anti-TCMR therapy (e.g., drug dosing for steroid pulse or lymphocyte depleting agents, steroid tapering and maintenance immunosuppression) as well recommendations for follow-up care are needed.

The survey demonstrates that borderline changes in indication and protocol biopsies are treated as rejection in most centers worldwide challenging the Banff classification and regulatory assessment [1], who do not consider borderline as rejection. Given the frequency of borderline changes, who have limited interobserver reproducibility and may depend on pathologist’s experience, one could speculate that eventually too many patients are treated. Randomized interventional trials are desperately needed for borderline lesions as well as objective tools (e.g., molecular diagnostics [13]) to identify those borderline changes, who benefit from treatment.

Interestingly, 36% of centers are performing regular protocol biopsies in Europe without clear evidence for a clinical benefit of this invasive procedure [9, 14, 15]. How to assess treatment response in patients with stable graft function? Undoubtedly, protocol biopsies are useful for clinical research, but there is a definitive need for good clinical trials to demonstrate improved outcomes after protocol biopsies.

Today, TCMR is frequently detected in “surveillance” biopsies due delayed or slow graft function in marginal kidneys. Tubulointerstitial infiltrates are found together with other pathologies such as acute tubular necrosis, capillaritis, or sclerotic lesions, making it difficult to differentiate inflammation or rejection from other causes of graft dysfunction. The classical case of an isolated TCMR several weeks after transplant with rising creatinine and quick response to treatment has become rare under current immunosuppression. Today’s pathology conference is characterized by mixed pathologies in marginal kidneys with delayed/slow function making it difficult to assess an adequate treatment response without “baseline” values. These complexities may explain some of the heterogeneity in the survey and we need better ways to define treatment response in patients with mixed pathologies with or without delayed graft function. Granular data on the evolution of renal function and on the molecular and histological resolution of TCMR are desperately needed. Future research and clinical trials for TCMR should include follow-up biopsies and innovative biomarkers to improve our understanding of TCMR.

In summary, the European survey provides important information on current practice for diagnosis and treatment of TCMR, identifies current limitations and unmet medical needs and calls for action to solve these fundamental problems after kidney transplantation.
